# Editorial: Care during pregnancy and early childhood for growth and development in low- and middle- income countries

**DOI:** 10.3389/fnut.2023.1361926

**Published:** 2024-01-09

**Authors:** Ranadip Chowdhury, Ravi Prakash Upadhyay, Bireshwar Sinha, Sunita Taneja, Jai K. Das, Nita Bhandari

**Affiliations:** ^1^Society for Applied Studies, New Delhi, India; ^2^Division of Women and Child Health, Aga Khan University, Karachi, Pakistan

**Keywords:** antenatal care, mental health, postnatal care, breastfeeding, complementary feeding, child stimulation, low birth weight

Enhanced efforts have been made in recent years to improve access and utilization of optimal care practices during pregnancy and early childhood in low- and middle-income countries (LMICs) (1). Yet only around half of the pregnant women receive the minimum of the four recommended antenatal visits and this has led to a high burden of adverse pregnancy outcomes, such as babies born with low birth weight, small-for-gestational age, or preterm (2). Postnatal care and care in the first 2 years of life are also suboptimal, resulting in a high burden of undernutrition and the child's inability to achieve optimal neuro-cognitive development. There are numerous interventions for each lifecycle stage which can improve maternal and child health, and nutritional status (3).

## Pregnancy care

In this supplement various important aspects of pregnancy care in low- and middle- income countries (LMICs) have been considered. The World Health Organization recommends eight antenatal care visits during pregnancy as a key strategy to promote women's health (4). Evidence form LMICs suggests that women who have had eight or more antenatal visits were more likely to have deliveries in health facilities and early postnatal care seeking (Chilot et al.).

Maternal undernutrition during pregnancy is another important issue that remains unaddressed in LMICs. A study in Ethiopia showed around half (45%) of the pregnant women were undernourished and key factors include lack of women's decision-making autonomy, household food insecurity, lack of prenatal dietary advice, poor hand washing and lack of proper sanitation (Arero). Nausea and vomiting in early pregnancy can hamper maternal food intake and impact mothers' nutritional status. A systematic review and metanalysis included in this supplement summarizes the available evidence on efficacy of complementary and alternative medicine therapies, including acupuncture, acupressure, and ginger in comparison with first-line medications or placebo for management of nausea and vomiting during pregnancy and suggested that these interventions though showed promise but quality of evidence is low (Tan et al.).

Prenatal screening and pre-pregnancy care is of utmost importance for healthy pregnancy and the case study from Mexico discusses a care model which combines data from maternal characteristics and history, laboratory tests in early gestation (11–13 weeks) to identify patient-specific risks for pregnancy complications and fetal loss and to improve equity (Bermudez Rojas et al.).

Maternal mental health has increasingly been recognized as a vital component of antenatal care. A study from West Ethiopia, shows approximately one-fifth of women with symptoms of depression during the antenatal period and the factors include being single, unplanned pregnancy, and dissatisfactory relationship with a partner (Oljira et al.). Hence recognizing and addressing mental health concerns during antenatal care is essential for comprehensive support to expectant mothers and interventions should focus on social and emotional factors.

## Child growth

The supplement offers valuable insights into the stunting determinants, postnatal growth in preterm infants, and nutrition-infection interactions during the initial 5 years of life. A study from Indonesia highlights the need for efficient and effective intervention programs targeting women's nutritional status during childbearing age and educating on child feeding practices to prevent stunting in children aged 6–23 months (Kartasurya et al.). Another study from Pakistan identified associated growth indicators at around 5 years with poverty, inappropriate complementary feeding, and infections during the first year (González-Fernández et al.). 'Catch-up growth' is identified as a potential modifier of early programming effects, especially for preterm and term SGA babies, predicting optimum neurodevelopment in later life but preterm babies in LMICs face challenges in achieving catch-up growth (5–8). A study from Indonesia reveals that preterm infants with postnatal growth failure experienced delayed full oral feeding and prolonged total parenteral nutrition (Rohsiswatmo et al.). A systematic review of probiotics suggested low to very low certainty of evidence of impact on reducing mortality and necrotizing enterocolitis (Thomas et al.).

## Breastfeeding and complementary feeding practices

Optimal breastfeeding and complementary feeding practices form the foundation of child growth and development (9). The COVID-19 pandemic disrupted these practices and a study in India highlighted that separation of mothers and newborns led to disruptions in breastfeeding practices within health institutions (Maria et al.). Expressed breast milk, preferably from the biological mother, is the best alternative for premature and small for gestational age babies and a study from India demonstrated that breastfeeding counseling interventions, including expressed breastfeeding and lactation management in the first 6 months, achieved 70% exclusive breastfeeding in these vulnerable infants (Sharma et al.). A desk review and key informant interviews in seven South Asian countries revealed that integrating maternal, infant, and young child nutrition (MIYCN) counseling indicators into the country's health information systems can positively impact behaviors related to MIYCN (Bhanot et al.).

## Early child development

The supplement included rigorous original studies for factors that could potentially influence child development in the early formative years. The study from Vietnam found no association between maternal hemoglobin (Hb) concentrations during pregnancy and various child health and development outcomes (Young et al.). These findings are in contrast with previous examinations that have shown maternal Hb to be associated with infant and child neurodevelopment and brain structural development. These contradictions highlight the need for further research to understand these relations particularly in resource-poor settings (10, 11). Children exposed to environments with lack of optimal stimulation, responsive care and learning opportunities are at risk for poor cognitive and developmental outcomes (12, 13). Maternal bonding and quality of time spent with a child are considered crucial for child's development. A study from rural India indicated that mothers dedicated a significant part of their day to household chores and about one-sixth time focused on childcare during infancy and as the child grew, maternal time shifted to income-generating and leisure activities (Batura et al.). This shift poses challenges in resource-poor households, suggesting difficulties in promoting and optimal caregiving behaviors.

The Sustainable programme incorporating nutrition and games (SPRING) intervention trial was implemented through monthly home visits by community-based workers in rural India and Pakistan and aimed to enhance early child development (ECD) and growth, but there no significant effects on ECD outcomes or growth in either setting (Kirkwood et al.). The investigators attributed the lack of overall impact to implementation shortcomings, as the challenges of integrating additional tasks into community worker's workload without sufficient resources and highlighted the potential suitability of the non-governmental organization model for scale-up, emphasizing the need for robust administrative and management systems to support successful implementation.

## Conclusions

This supplement sheds light on various facets of pregnancy and early childhood care, emphasizing the global need for increased efforts to enhance maternal and child health outcomes. It delves into the intricate interplay of maternal health - both physical and mental environmental factors, and the difficulties associated with implementing interventions in resource-poor settings. Notable challenges include inadequate antenatal care in LMICs, unaddressed maternal undernutrition, and limited access to intervention programs for prevention of stunting in young children. The interplay of maternal health with child development and challenges faced by mothers in resource-poor households also requires deeper exploration. Implementation challenges, as highlighted by the SPRING trial, call for enhanced field supervision, skill development, and workload management. Bridging these gaps requires concerted efforts, from improving antenatal care and maternal nutrition to addressing issues related to access to quality childhood nutrition and care related interventions.

## Author contributions

RC: Writing—original draft, Writing—review & editing. RU: Writing—original draft, Writing—review & editing. BS: Writing—original draft, Writing—review & editing. ST: Writing—original draft, Writing—review & editing. JD: Writing—original draft, Writing—review & editing. NB: Writing—original draft, Writing—review & editing.
